# 

**Published:** 1864-08

**Authors:** 


*Contents.*

ORIGINAL COMMUNICATIONS .......................................... 113

1. Epidemic Cerebro-Spinal Meningitis, by Surgeon G. A. Moses. 2. Report of Surgical Cases in General Hospital, Fayetteville, North Carolina, by Surgeon Benjamin F. Fessenden. 3. Veratrum Viride in Acute Mania a Potu, by C. R. Harris, M. D. 4. Excision of the Right-Superior Maxilla, and of a considerable portion of the Left, for a Tumor of the Antrum, by E. R. Mordecai, M. D. 5. Resection of Superior-Third of Humerus, by Asst. Surg. F. Marion Letcher. 6. Saline Treatment of Acute Dysentery, by Surgeon Jas. Bolton.

EDITORIAL .......................................... 119

External Application of Turpentine as a substitute for the internal use of Quinine in the treatment of Intermittent Fever.

CHRONICLE OF MEDICAL SCIENCE .......................... 120

Motion of Balls, and Ball Wounds, translated from the French of Legouest. On the Ordeal Bean of Calabar; its action on the Human Body, compared with that of Woorara and Conia. Popliteal Aneurism successfully treated by the flexion of the limb, after a failure of treatment by compression, by S. Curey, M. D., C. B., Deputy Inspector-General of Hospitals.

INTERESTING ANALYSES OF ARTICLES IN FOREIGN JOURNALS .......................................... 125

